# Epiplooplastie médiastinale dans le traitement d'une médiastinite post chirurgie cardiaque: à propos d'un cas

**DOI:** 10.11604/pamj.2014.19.308.5596

**Published:** 2014-11-21

**Authors:** Hicham Labsaili, Waseem Borik, Pierre Demondion, Pascal Leprince

**Affiliations:** 1 Service de Chirurgie Thoracique et Cardio-Vasculaire, Institut du Cœur, Hôpital de la pitié salpêtrière, Paris, France

**Keywords:** Médiastinite, épiplooplastie, infection du site opératoire, Mediastinitis, omental flap, surgical site infection

## Abstract

Nous rapportons le cas d'un patient diabétique et obèse, opéré d'une chirurgie de revascularisation myocardique avec l'utilisation des deux artères mammaires internes comme greffon. L'intervention s'est compliquée d'une médiastinite traitée premièrement à thorax fermé et secondairement par une épiplooplastie. Cette technique reste efficace dans les médiastinites graves et délabrantes. Tout chirurgien cardiaque et digestif doit la connaitre.

## Introduction

La médiastinite aigue est une complication infectieuse grave du site opératoire survenant au décours immédiat d'une chirurgie cardiaque réalisée par sternotomie. Elle complique environ 2% des interventions [1]. Cette complication est responsable d'une prolongation de l'hospitalisation et d'une mortalité élevée d'où la nécessité de la traiter en urgence. Le traitement comporte un volet médical et un autre chirurgical. Parmi les techniques chirurgicales utilisées: le recouvrement du médiastin antérieur à l'aide de l’épiploon. Nous rapportons ici le cas d'un patient traité avec succès par cette technique.

## Patient et observation

**Figure 1:**
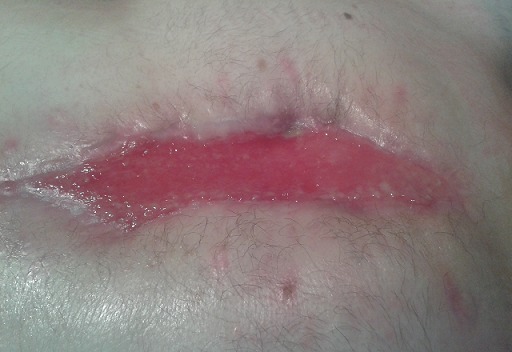
Cicatrisation complète de l'epiplooplastie après un mois de l'intervention

Il s'agit d'un patient de 65 ans, diabétique, obèse et hypertendu, opéré pour un quadruple pontage coronarien tout artériel, avec l'utilisation des deux artères mammaires internes qui a provoqué peut être une dévascularisation de la face postérieure du sternum. Dans le 8^ème^ jour après l'intervention, le patient a ressenti une douleur à la mobilisation des berges sternales et un aspect inflammatoire de la cicatrice avec un écoulement plus ou moins louche. Le patient a été repris au bloc opératoire pour une cure de médiastinite dite à thorax fermé. L’évolution a été défavorable avec l'apparition d'une disjonction sternale, une instabilité des berges et une douleur spontanée. L'ouverture de la cicatrice a été réalisée et le médiastin antérieur est laissé ouvert en cicatrisation dirigée. Cette exposition sternale présente de nombreux inconvénients: risque de rupture de pontage, de gros vaisseaux ou de la paroi myocardique, mettant en jeu le pronostic vital de manière immédiate. Pour minoriser ces risques, on a eu recours à un recouvrement du médiastin par de l’épiploon. L’évolution a été bien favorable, le patient a cicatrisé un mois plus tard (Figure 1).

### Technique opératoire

**Figure 2:**
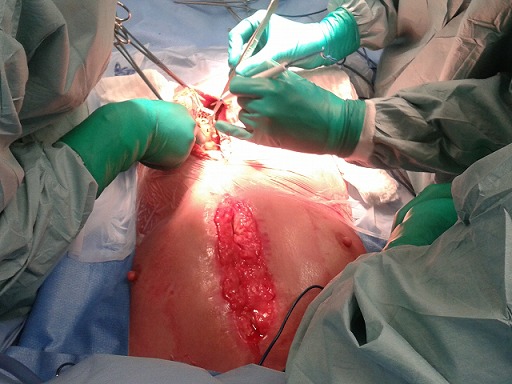
Prélèvement de l’épiploon après une laparotomie sus ombilicale laissant un pont cutané

**Figure 3:**
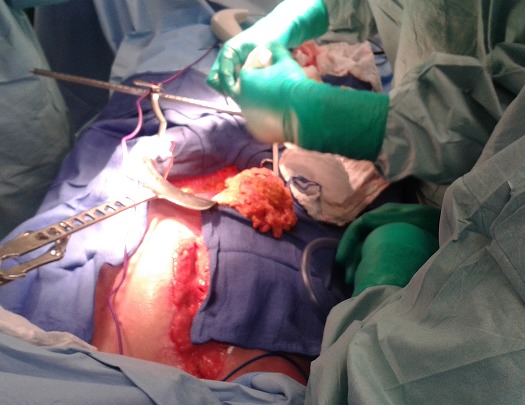
Pédiculation de l’épiploon et soulèvement du tablier épiploique

**Figure 4:**
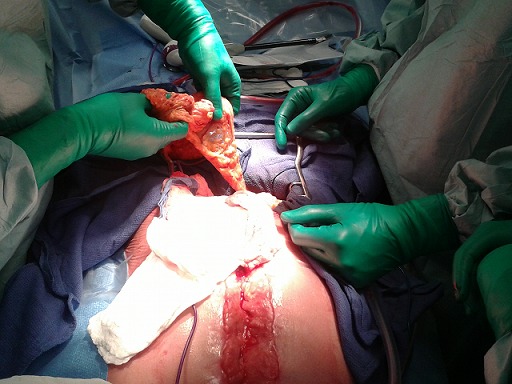
Tunnelisation de l’épiplooplastie et sa fixation sur le sternum délabré

Le patient est installé en décubitus dorsal, avec thorax et abdomen dans le champ opératoire. On procède par la laparotomie médiane afin de prélever l’épiploon (Figure 2). Une incision médiane sus-ombilicale essaie de préserver un pont cutané entre l'ouverture médiastinale et l'incision médiane ombilicale. La pédiculation de l’épiploon sur l'artère gastroépiploique droite est idéale, offrant le trajet le plus direct vers le médiastin, puis le décollement colo-épiploique est fait en soulevant le tablier épiploique vers le haut (Figure 3). Après avoir sectionnée l'artère gastroépiploique gauche, on a pédiculisé la plastie sur l'artère gastroépiploique droite en sectionnant au ras de la séreuse gastrique chaque vaisseau en respectant l'arcade de la grande courbure gastrique. Un tunnel rétro-xiphoidien créé au doigt a permis l'ascension de l’épiplooplastie dans le médiastin à l'aide d'une pince longuette, en prenant garde de ne pas twister les pédicules vasculaires (Figure 4). L'epiplooplastie est fixé aux berges de la plaie médiastinale par des points séparés de fils résorbables. Un pansement à base de tulle gras et compresses est fait en dernier.

## Discussion

Les médiastinites post chirurgicales sont des infections graves. Elles peuvent être classées suivant leur localisation médiastinale, mais elles le sont le plus souvent selon leur siège antérieur ou postérieur. En chirurgie cardiaque, ce sont les médiastinites antérieures qui surviennent. Le diagnostic d'infection sternale profonde nécessite une prise en charge chirurgicale précoce au bloc opératoire, les approches chirurgicales dépendent des habitudes chirurgicales locales et ont évolué au cours du temps pour une même équipe [2]. L'aspiration drainage à thorax fermé s'est cependant généralisée comme traitement initial standard. En cas d’échec de cette technique ou bien en cas d'une médiastinite d'emblée délabrante, le recours à la mise à plat avec pansement à thorax ouvert avec plastie ultérieure à l'aide de l’épiploon s'impose. La technique d'aspiration drainage à thorax fermé est la plus simple à réaliser quand le diagnostic est fait précocement et que les dégâts locaux sont limités. Après évacuations des collections et excision des tissus infectés, plusieurs drains multi perforés de petit calibre sont placés dans la cavité médiastinale, entre les berges sternales, voir en sous-cutané et dans les plèvres si nécessaire afin de drainer parfaitement toutes les zones suspectes d'infection [3].

Nous avons utilisé cette technique chez notre patient en premier, mais devant la non amélioration vu les facteurs de risque associés. Nous avons eu recours aux techniques de fermeture à thorax ouvert. Cette stratégie se justifie surtout lorsqu'il existe une perte de substance importante [4], avec un sternum en partie détruit par l'infection, rendant une fermeture simple sans tension impossible. Le premier temps correspond à la réalisation de pansements à ciel ouvert. Le second temps, dès que l’état de la plaie le permet, consiste à refermer le sternum par une plastie. Les techniques faisant appel à la chirurgie de recouvrement comportent soit des lambeaux musculaires, soit une épiplooplastie. Le muscle le plus utilisé est le grand pectoral en présternal [5]. L’épiplooplastie est également largement utilisée [6]. Bien qu'il ne soit pas possible de porter des conclusions définitives, il semble que, à condition que le chirurgien ait une bonne expérience de la chirurgie abdominale et thoracique, les résultats de l’épiplooplastie soient supérieurs en termes de guérison locale et de survie.

## Conclusion

La prise en charge des médiastinites aiguës post chirurgie cardiaque a beaucoup évolué grâce à une meilleure connaissance de l’épidémiologie et de la prophylaxie de cette infection. La mortalité a en effet significativement diminué et est liée le plus souvent au terrain. Le recours aux techniques de plastie semble associé à un meilleur pronostic à moyen et long terme.
